# The long-term trend of Parkinson’s disease incidence and mortality in China and a Bayesian projection from 2020 to 2030

**DOI:** 10.3389/fnagi.2022.973310

**Published:** 2022-09-15

**Authors:** Fangyao Chen, Shiyu Chen, Aima Si, Yaqi Luo, Weiwei Hu, Yuxiang Zhang, Jiaojiao Ma

**Affiliations:** ^1^Department of Epidemiology and Biostatistics, School of Public Health, Xi’an Jiaotong University Health Science Center, Xi’an, China; ^2^Department of Radiology, First Affiliated Hospital of Xi’an Jiaotong University, Xi’an, China; ^3^Department of Neurology, Xi’an Gaoxin Hospital, Xi’an, China

**Keywords:** Parkinson’s disease, China, mortality and incidence, age-period-cohort, projection analysis

## Abstract

**Background:** Parkinson’s disease is a disabling degenerative disease of the central nervous system that occurs mainly in elderly people. The changes in the incidence and mortality of Parkinson’s disease at the national level in China over the past three decades have not been fully explored.

**Methods:** Research data were obtained from the Global Burden of Disease 2019 study. The trends of crude and age-standardized incidence and mortality rates by gender of Parkinson’s disease in China were analyzed with the age-period-cohort model and the Joinpoint regression analysis. The effects of age, time period, and birth cohort on the incidence and mortality of Parkinson’s disease were estimated. The gender- and age-specific incidence and mortality rates of Parkinson’s disease from 2020 to 2030 were projected using the Bayesian age-period-cohort model with integrated nested Laplace approximations.

**Results:** From 1990 to 2019, the annual percentage change of the age-standardized incidence rate was 0.8% (95% CI: 0.7%–0.8%) for males and 0.2% (95% CI, 0.2–0.2%) for females. And the age-standardized mortality rate for males was 2.9% (95% CI: 2.6%–3.2%) and 1.8% (95% CI: 1.5%–2.1%) for females. The results of the age-period-cohort analysis suggested that the risk and burden of Parkinson’s disease continued to increase for the last several decades. Projection analysis suggested that the overall Parkinson’s disease incidence will continue to increase for the next decades. It was projected that China would have 4.787 million Parkinson’s patients by the year 2030, however, the mortality of Parkinson’s disease for both genders in China may keep decreasing.

**Conclusion:** Though the mortality risk may decrease, Parkinson’s disease continues to become more common for both genders in China, especially in the senior-aged population. The burden associated with Parkinson’s disease would continue to grow. Urgent interventions should be implemented to reduce the burden of Parkinson’s disease in China.

## Introduction

Parkinson’s disease (PD) is a progressive neurodegenerative disease that occurs mainly in elder people (Aarsland et al., 2021). Typical symptoms of PD include resting tremors, muscle rigidity, slow movement, accompanied by mental disorder, dementia, and memory impairment (Beaton et al., 2022). Due to its motor symptoms, it is associated with a high disability rate (Fabbri et al., 2022). Usually, as the PD progresses, patients will gradually lose their ability to take care of themselves and eventually become bedridden (Fabbri et al., 2022). And PD mortality is mainly associated with the complications after being bedridden, such as repeated lung infections, nutritional disorders caused by difficulty eating, and others (Moscovich et al., 2017; Fabbri et al., 2022; Gonzalez et al., 2022). There are also some patients who fall and suffer fractures during activities and even suffer from a traumatic cerebral hemorrhage, which leads to a sharp decline in their health state (Nguyen et al., 2022).

China is currently the most populous country in the world. With the aging of the population and the changes in the living environment, PD has gradually become a major health problem that threatens the health and quality of life of the elderly in China (Yi et al., 2022). The results of a survey conducted in three cities (Beijing, Xi’an, and Shanghai) in 2005 showed that the prevalence of PD among people over 65 years old in China reached 1,700/100,000, and was increasing with age (Zhang et al., 2005). Another meta-analysis published in 2014 pointed out that the population aged 80 years and above has the highest prevalence of PD (about 1,663/100,000) in China (Zou et al., 2015). Although epidemiological investigations focusing on PD conducted in China during the past several decades have come to different conclusions, the results of six large-scale surveys conducted from 2001 to 2017 all suggested that the prevalence of PD in China in recent years was already significantly higher than that in the 1980s (Zhang et al., 2021). It was estimated that there would be more than 2 million PD patients in China by now, and this number will continue to rise (Winkler et al., 2010).

Since PD affects motor function, though the progress of the disease is not fast, most PD patients will eventually be confined at home or in hospital beds, thus becoming a burden to the family and society. In China, the average annual cost of PD was 3,225.94 USD in 2015 (Li et al., 2019). The direct and indirect costs caused by PD were 2,503.46 USD and 722.48 USD per capita (according to the exchange rate of RMB to USD in 2015) respectively (Li et al., 2019), while the GDP of China in 2015 was about 8,016 USD per capita. An epidemiological study conducted in China also found that the annual cost for the most serious or late-stage PD patients was almost five to six-times of that for the mild or early-stage ones (Liu et al., 2016). With the intensification of population aging, it is possible that China could become the country with the largest number of PD patients, and this would bring a heavy economic burden to the family and society.

The age-period-cohort (APC) model is a statistical model that estimates the effects of age, year period, and birth cohort on the incidence of an outcome variable (Bell, 2020). With literature review, we found there are few studies focused on the long-term trend of PD incidence and mortality in China, and corresponding projection. Besides, most of the existing studies are limited to some provinces or within a short time span. Therefore, in this study, we used the APC model and Joinpoint regression model to analyze the long-time trend of PD incidence and mortality in Chinese residents from 1990 to 2019 and estimate the effects of age, time period, and birth cohort on PD risk. Then, we applied the Bayesian age-period-cohort (BAPC) model to project the incidence and mortality of PD in China from 2020 to 2030. We aimed to provide valuable quantitative evidence for the prevention and control of PD in China and the management of medical resources.

## Materials and Methods

### Data source and extraction

This study was conducted based on the summarized data obtained from the Global Burden of Disease (GBD) 2019 study^1^. The GBD 2019 study provides comprehensive quantitative statistics of 369 types of diseases and injuries and 87 risk factors in 204 countries and territories (GBD 2019 Diseases and Injuries Collaborators, 2020).

During the data extraction process, we set the location of the extracted data as China in the data extraction interface in the GBD 2019 database^1^, and the measure was defined as death and incidence. The cause is set as Parkinson’s disease. The summarized data available in the GBD database are organized in grouped into 5-year groups. Considering the age- and gender-specific features of PD, We selected sex-disaggregated summarized incidence and mortality data.

### Main outcome and measurements

The main outcome is the incidence and mortality rates of PD from 1990 to 2019 in China. The definition of PD in the GBD 2019 study was according to the International Statistical Classification of Diseases and Related Health Problems, Ninth Revision (ICD-9; World Health Organization, 2021a) or Tenth Revision (ICD-10; World Health Organization, 2021b).

### Statistical analysis

We used the APC model to estimate the effects of age, year period, and birth cohort. To convert the data to meet the requirement of the APC model, we re-organized the data into consecutive 5-year periods from 1990 to 2019 and 5-year age groups from the age of 25–29 years old to 90–94. Since the incidence of PD under age 25 is extremely low, we removed the age groups under 25 years old. The data in the age group above 95 were also excluded since the GBD 2019 data could not verify the exact age after the age of 95 years old. The estimation of the APC model was conducted through the APC Web Tool^2^ (Rosenberg et al., 2014).

The Joinpoint regression analysis was performed to estimate the long-time non-linear changes in PD incidence and mortality and corresponding gender difference from 1990 to 2019 in China. The long-time changes were evaluated using the average percent changes (AVPCs) and average annual percent changes (AAPCs). The Joinpoint regression was conducted through the Joinpoint Regression Program Software released by the National Cancer Institute of US (version 4.9.0.0, March 2021; Statistical Research and Application Branch, NCI, USA).

We projected the age-specific PD incidence and mortality from 2020 to 2030 by gender in China. The projection analysis was conducted using the Bayesian age-period-cohort (BAPC) model (Riebler and Held, 2017). The basic age-period-cohort model can be defined as Newman (2002):


$$R_{ijk}=\mu +\alpha_{i}\;Age+\beta_{j}\;Period+\gamma_{k}$$

Among which, *μ* is the constant, *R_ijk_* represents the incidence or mortality rate of PD in the *i*th age group, *j*th time period, and kth birth cohort. *α_i_*, *β_j_*, and *γ_k_* are the effects of age, time period, and birth cohort. Classical APC models use sample statistics to estimate parameters, while the BAPC model can utilize both sample information and prior information (Schmid and Held, 2007). The prior information utilized in the BAPC model mainly refers to the prior probability distribution of time period and birth cohort effects, and the model estimates the effects of age, period, and cohort through a random walk of different orders (Schmid and Held, 2007; Riebler and Held, 2017). The effects of age in a BAPC model can be estimated as (Schmid and Held, 2007; Riebler and Held, 2017):


$$f\left(\alpha |l_{\alpha }\right)\alpha_{l_{\alpha }}^{\left(I-2\right)/2}\exp\left[-\frac{l_{\alpha }}{2}\sum_{i=3}^{I}{\left(\alpha_{i}-2\alpha_{i-1}+\alpha_{i-2}\right)}^{2}\right]$$

In which, *l*_α_ represents the variance parameter. An independent random effect $z_{ij}∼N\left(0,k_{z}^{-1}\right)$ is also added into the model for ith age group at time period *t* for the following years to avoid over dispersion, as:


$$R_{ijk}=\mu +\alpha_{i}\;Age+\beta_{j+t}\;Period+\gamma_{k+t}\;Cohort+z_{ij+t}$$

Then, the effects of the time period in (*j* + 1)th period can be assumed to follow the distribution as:


$$\beta_{j+1}|\beta_{1},...\beta_{j},k_{\beta }∼N\left(2\beta_{j}-\beta_{j-1},k_{\beta }^{-1}\right)$$

The BAPC model uses the Markov chain Monte Carlo method to estimate parameters. The more iterations, the more accurate the model estimation. In this study, the time of iteration was set to be 10,000. The estimation of the model was based on integrated nested Laplace approximations using R-programming language (version 4.0.3) and RStudio (Version 1.1.463, RStudio Inc., MA, USA) with R packages “BACP” (Riebler and Held, 2017), “BAMP” (Schmid and Held, 2007), and “INLA” (Lindgren and Rue, 2015). A *P*-value less than 0.05 was considered statistically significant.

## Results

### Long-time trend and projection of PD incidence

#### APC analysis for PD incidence

**Figure 1:**
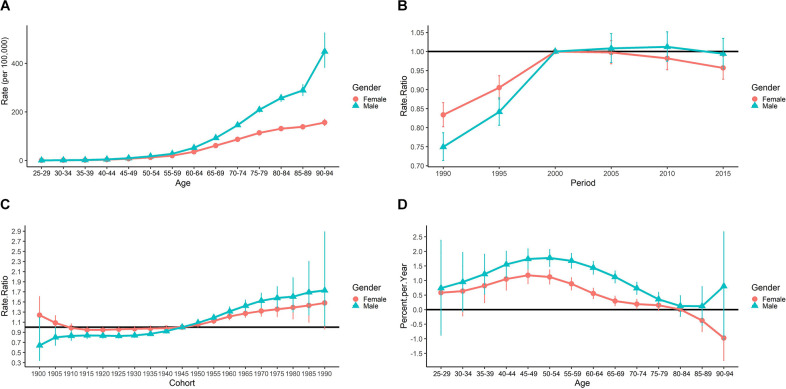
Results of APC analysis for PD incidence from 1990 to 2019. **(A)** Longitudinal age curve of PD incidence (per 100,000) by gender. **(B)** Period RR by gender. **(C)** Cohort RR by gender. **(D)** Local drifts by gender.

The longitudinal age curve of PD incidence was presented in Figure 1A. Overall, the PD incidence (per 100,000) shows an increasing trend along with age groups for both genders. We also found that before the age group of 55–59, the gender difference is not large, however, the increasing trend for males in China is more rapid than that of females thereafter. For males, the PD incidence rate increased from 0.321 per 100,000 to 449.030 per 100,000 populations from the age group of 25–29 to 90–94. For females, the incidence rates increased from 0.254 per 100,000 to 156.003 per 100,000 from the age group of 25–29 to 90–94.

The estimated period RRs and gender differences were shown in Figure 1B. The period RRs for males in China showed an up-and-stable trend. The reference year was the year 2000, while for females the trend was up-then-down. The estimated period RRs for males after the year 2000 was not statistically significant (*P* > 0.05). But for females, the estimated period RRs were all lower than 1 and was statistically significant in the year 2015 (*P* < 0.05).

The estimated cohort RRs by gender were shown in Figure 1C. The cohort RRs for both genders showed a significant upward trend across birth cohorts. The increasing trend for females was higher than that of males after the year 1945, while before that the estimated cohort RRs for females was higher than that of males.

The local drifts of the PD incidence for both genders in China were presented in Figure 1D. Generally speaking, the local drifts for males and females showed different trends. As shown in Figure 1D, the local drifts for males and females in China both showed an upward trend (above 0) before age groups of 50–54, while then decreases till age group 80–84. Then, the local drifts for males increased again while that for females continued to decrease. The overall net drift is 1.135% (95% CI: 0.938%–1.333%; *P* < 0.001) for males, and 0.534% (95% CI: 0.381%–0.687%; *P* < 0.001) for females.

#### Joinpoint analysis for PD incidence

The result of the Joinpoint analysis for PD incidence was presented in Table 1. There are five trends for the crude incidence rates (CIRs) of females in China, with an average annual percent change (AAPCs) of 2.9% (95% CI: 2.9%–2.9%, *P* < 0.001) per year from 1990 to 2019. The peak of growth appeared in Trend 2 from 1995 to 1999 with 4.4% (95% CI: 4.2%–4.6%, *P* < 0.001) per year. For the CIRs of males in China, the AAPCs is 3.5% (95% CI: 3.5%–3.6%). The peak of growth appeared in Trend 2 from 1995 to 1999 with 6.5% (95% CI: 6.3%–6.7%, *P* < 0.001) per year.

**Table 1:** Average and annual percent changes of PD incidence in China from 1990 to 2019.

		**CIRs**				**ASIRs**		
		**Time period**	**APC (95% CI)**	***t*(P)**	**Time period**	**APC (95% CI)**	***t*(P)**
Female	Trend 1	1990–1995	2.9* (2.8, 3)	60.3 (< 0.001)	1990–1995	0.9* (0.8, 0.9)	33.6 (< 0.001)
	Trend 2	1995–1999	4.4* (4.2, 4.6)	48.5 (< 0.001)	1995–1999	1.7* (1.6, 1.8)	31.8 (< 0.001)
	Trend 3	1999–2007	2.3* (2.3, 2.4)	116.5 (< 0.001)	1999–2002	0.2 (0.0, 0.4)	1.8 (0.091)
	Trend 4	2007–2017	2.7* (2.7, 2.8)	250.5 (< 0.001)	2002–2012	-0.3* (-0.3, -0.3)	-32.5 (< 0.001)
	Trend 5	2017–2019	3.1* (2.9, 3.3)	30.9 (< 0.001)	2012–2017	-0.5* (-0.5, -0.4)	−13 (< 0.001)
	Trend 6	-	-	-	2017–2019	-0.1 (-0.4, 0.1)	-1.1 (0.302)
	AAPC	1990–2019	2.9* (2.9, 2.9)	163.9 (< 0.001)	1990–2019	0.2* (0.2, 0.2)	12.3 (< 0.001)
Male	Trend 1	1990–1995	3.7* (3.6, 3.8)	69 (< 0.001)	1990–1996	1.7* (1.7, 1.8)	105.3 (< 0.001)
	Trend 2	1995–1999	6.5* (6.3, 6.7)	67.8 (< 0.001)	1996–1999	3.2* (3.0, 3.4)	36.7 (< 0.001)
	Trend 3	1999–2006	2.9* (2.9, 3)	116.3 (< 0.001)	1999–2002	0.5* (0.4, 0.7)	6.5 (< 0.001)
	Trend 4	2006–2009	3.4* (3.1, 3.6)	25.9 (< 0.001)	2002–2009	0.3* (0.2, 0.3)	19.4 (< 0.001)
	Trend 5	2009–2017	2.9* (2.9, 2.9)	198.2 (< 0.001)	2009–2017	0.1* (0.1, 0.1)	7.6 (< 0.001)
	Trend 6	2017–2019	2.1* (1.9, 2.3)	22.7 (< 0.001)	2017–2019	-0.7* (-0.9, -0.6)	-9.1 (< 0.001)
	AAPC	1990–2019	3.5* (3.5, 3.6)	153.1 (< 0.001)	1990–2019	0.8* (0.7, 0.8)	53.3 (< 0.001)

*: statistical significant.

For the age-standardized incidence rates (ASIRs) of females, the AAPCs was 0.2% (95% CI: 0.2%–0.2%) per year. The ASIRs for females before 2017 were above 0, and then below 0 thereafter. The peak appeared in Trend 2 from 1995 to 1999 with 1.7% (95% CI: 1.6%–1.8%, *P* < 0.001). For males, the APCs were above 0 before the year 2002, while thereafter the AAPCs were below 0, the AAPCs was 0.8% (95% CI: 0.7%–0.8%). The peak of growth appeared in Trend 2 from 1996 to 1999 with 3.2% (95% CI: 3.0%–3.4%, *P* < 0.001) per year for males as presented in Table 1.

#### Bayesian projection of PD incidence

**Figure 2:**
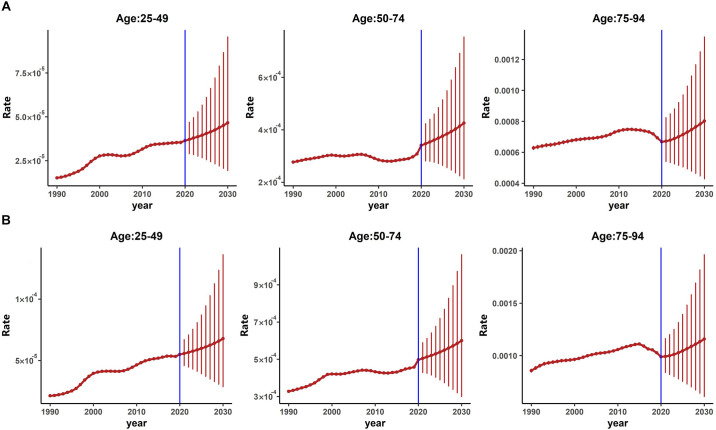
Projected age-specific incidence of PD from 2020 to 2030 by gender. **(A)** Projected age-specific PD incidence for females. **(B)** Projected age-specific PD incidence for males.

As shown in Figure 2A (females) and Figure 2B (males), our results indicated that for both genders, the overall PD CIRs in China would continue to increase from 2020 to 2030. The projected trends of CIRs for both genders were similar, however, the projected CIRs for males were higher than that of females in almost all three age groups.

Table 2 presented the projected numbers of PD patients from 2020 to 2030. We could see that the projected number of PD patients showed an upward trend. The number of projected male PD patients was more than that of females. The number of PD patients was projected to reach about 4.787 million in 2030 in China in total.

**Table 2:** Projected age-specific number of PD patients in China by gender from 2020 to 2030 based on the BAPC framework^a^.

**Year**	**Gender**	**Age Groups**			**Total**	
		**25–**	**30–**	**35–**	**Gender-specific**	**All**
	Female	8,477	82,082	43,375	133,933
2020						327,145
	Male	14,284	120,848	58,080	193,212
	Female	8,951	87,338	51,709	147,997
2021						367,377
	Male	14,261	131,285	73,834	219,380
	Female	9,020	89,104	55,177	153,302
2022						378,984
	Male	14,359	133,306	78,017	225,683
	Female	9,029	91,645	58,775	159,449
2023						392,602
	Male	14,401	136,441	82,312	233,153
	Female	9,116	93,682	63,051	165,849
2024						406,845
	Male	14,508	138,801	87,687	240,996
	Female	9,106	94,708	68,808	172,621
2025						423,928
	Male	14,918	139,832	96,557	251,307
	Female	9,396	98,986	77,991	186,372
2026						461,484
	Male	15,013	146,899	113,200	275,112
	Female	9,464	101,914	83,323	194,702
2027						480,034
	Male	15,151	150,308	119,874	285,333
	Female	9,572	105,353	88,090	203,015
2028						499,174
	Male	15,398	154,414	126,346	296,159
	Female	9,051	100,498	98,731	208,279
2029						514,008
	Male	15,702	159,498	130,529	305,730
	Female	8,382	107,485	70,113	185,979
2030						478,744
	Male	13,014	169,970	109,780	292,765

a: The predicted values are rounded to retain only the integer portion.

### Long-time trend and projection of PD mortality

#### APC analysis for PD mortality

**Figure 3:**
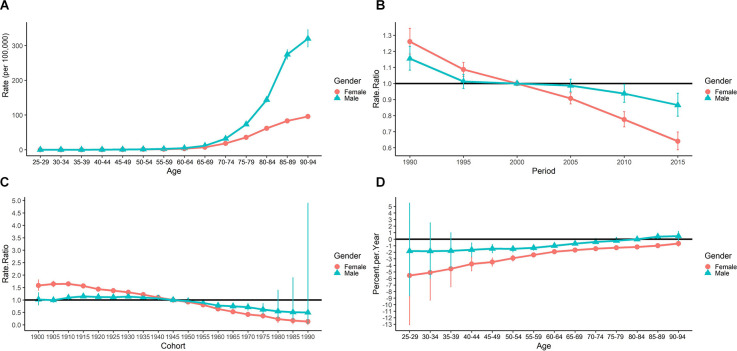
Results of APC analysis for PD mortality from 1990 to 2019. **(A)** Longitudinal age curve of PD incidence (per 100,000) by gender. **(B)** Period RR by gender. **(C)** Cohort RR by gender. **(D)** Local drifts by gender.

The longitudinal age curve of PD mortality was presented in Figure 3A. The PD mortality rates showed an increasing trend along with age groups for both genders. For males, the mortality rates increased from 0.009 per 100,000 to 320.109 per 100,000 from the age group of 25–29 to 90–94, while for females, it increased from 0.012 per 100,000 to 95.738 per 100,000. The gender differences were not significant before the age group 65–69, while thereafter, the mortality of males increased more rapidly than that of females.

The estimated period RRs by gender were shown in Figure 3B. The period RRs for both genders in China showed a downward trend. The reference year was the year 2000. The estimated period RRs for females was higher than that of males before the year 2000 (all above 1), however, the cohort RRs for males became higher after the year 2000 (all less than 1).

The estimated cohort RRs by gender were shown in Figure 3C. The cohort RRs for Chinese males and females both showed a significant downward trend across birth cohorts. We also found that for Chinese females the cohort RRs were higher than that of males before the year 1945, and then the RRs for males were higher.

The local drifts of the PD mortality for both genders in China were presented in Figure 3D. Generally speaking, the local drifts for males and females showed the same upward trends. And the local drifts for males were higher than that of females. The overall net drift was −0.96% (95% CI: −1.485% to −0.432%; *P* < 0.001) for males, and −2.537% (95% CI: −3.083% to −1.989%; *P* < 0.001) for females.

#### Joinpoint analysis for PD mortality

The result of the Joinpoint analysis for PD mortality rates was presented in Table 3. For the crude mortality rates (CMRs) of females in China, with an average increase (AAPCs) of 1.8% (95% CI: 1.5%–2.1%, *P* < 0.001) per year from 1990 to 2019. The peak of growth appeared in Trend 2 from 1998 to 2004 at 3.2% (95% CI: 2.7%–3.7%, *P* < 0.001) per year. For the CIRs of males in China, the AAPC is 2.9% (95% CI: 2.6%–3.2%). The peak of growth appeared in Trend 4 from 2007 to 2010 at 5.0% (95% CI: 3.4%–6.7%, *P* < 0.001) per year.

**Table 3:** Average and annual percent changes of PD mortality in China from 1990 to 2019.

		**CMRs**			**ASMRs**		
		**Time period**	**APC (95% CI)**	***t*(P)**	**Time period**	**APC (95% CI)**	***t*(P)**
Female	Trend 1	1990–1998	1.0* (0.7, 1.3)	7.4 (< 0.001)	1990–1998	-1.0* (-1.3, -0.8)	−9 (< 0.001)
	Trend 2	1998–2004	3.2* (2.7, 3.7)	13.6 (< 0.001)	1998–2004	0 (-0.5, 0.5)	0 (0.964)
	Trend 3	2004–2007	-0.8 (-2.7, 1.2)	-0.8 (0.416)	2004–2007	-3.6* (-5.8, -1.4)	-3.6 (0.004)
	Trend 4	2007–2010	2.8* (0.8, 4.9)	3.1 (0.009)	2007–2010	-0.4 (-2.8, 2.1)	-0.3 (0.759)
	Trend 5	2010–2015	0.6* (0, 1.2)	2.2 (0.044)	2010–2015	-2.9* (-3.7, -2)	-7.4 (< 0.001)
	Trend 6	2015–2019	3.8* (3.1, 4.4)	13.5 (< 0.001)	2015–2019	-0.2 (-1.2, 0.8)	-0.3 (0.732)
	AAPC	1990–2019	1.8* (1.5, 2.1)	11.1 (< 0.001)	1990–2019	-1.2* (-1.6, -0.8)	-6.3 (< 0.001)
Male	Trend 1	1990–1995	0.9* (0.3, 1.5)	3.3 (0.005)	1990–1994	-1.3* (-1.9, -0.7)	-4.7 (< 0.001)
	Trend 2	1995–2004	4.3* (4, 4.5)	39 (< 0.001)	1994–2004	0.5* (0.3, 0.7)	6.5 (< 0.001)
	Trend 3	2004–2007	1.6 (-0.1, 3.4)	2 (0.069)	2004–2007	-1.4 (-3.2, 0.5)	-1.6 (0.137)
	Trend 4	2007–2010	5.0* (3.4, 6.7)	6.8 (< 0.001)	2007–2011	1.5* (0.6, 2.4)	3.5 (0.003)
	Trend 5	2010–2019	2.4* (2.3, 2.5)	41.3 (< 0.001)	2011–2019	-1.4* (-1.6, -1.2)	-13.8 (< 0.001)
	AAPC	1990–2019	2.9* (2.6, 3.2)	22.2 (< 0.001)	1990–2019	-0.3* (-0.6, -0.1)	-2.7 (< 0.001)

*: statistical significant.

For the age-standardized mortality rates (ASMRs) of females, the AAPC was −1.2% (95% CI: −1.6% to −0.8%) per year. The peak appeared in Trend 3 from 2004 to 2007 at −3.6% (95% CI: −5.8% to −1.4%, *P* < 0.001). For the ASIRs of males in China, the AAPC was −0.3% (95% CI: −0.6% to −0.1%). The peak of growth appeared in Trend 4 from 2007 to 2011 at 1.5% (95% CI: 0.6%–2.4%, *P* < 0.001) per year.

#### Bayesian projection of PD mortality

**Figure 4:**
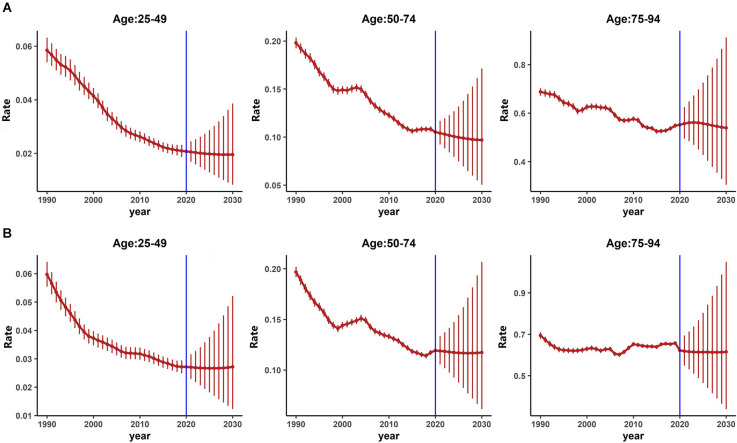
Projected age-specific mortality of PD from 2020 to 2030 by gender. **(A)** Projected age-specific PD mortality for females. **(B)** Projected age-specific PD mortality for males.

As shown in Figures 4A (females) and Figure 4B (males), the projected CMRs trends for both genders were not consistent across age groups from 2020 to 2030. For females, as in Figure 4A, the projected CMRs showed a downward trend for the age groups of 25–49 and 50–74, while for the age group 75–94, the projected CMRs showed a downward trend. However, for males as in Figure 4B, the projected CMRs showed a stable and slightly downward trend for all age groups.

Table 4 presented the projected numbers of PD mortality from 2020 to 2030 for three age groups. Though the projected number of PD mortality also showed an upward trend.

The results of the projected analysis suggested that males had higher projected mortality risks than females, and the risk increased along with age.

**Table 4:** Projected age-specific number of PD mortality in China by gender from 2020 to 2030 based on the BAPC framework^a^.

**Year**	**Gender**	**Age groups**			**Total**	
		**25–**	**30–**	**35–**	**Gender-specific**	**All**
	Female	168	8,424	23,204	31,796	
2020						82,328
	Male	354	14,849	35,329	50,532	
	Female	172	8,900	27,344	36,416	
2021						97,297
	Male	340	16,355	44,186	60,881	
	Female	170	9,089	28,805	38,064	
2022						101,330
	Male	338	16,821	46,107	63,266	
	Female	164	9,504	30,047	39,715	
2023						105,935
	Male	328	17,703	48,189	66,220	
	Female	158	9,712	31,370	41,240	
2024						110,439
	Male	319	18,194	50,686	69,199	
	Female	152	9,842	32,879	42,873	
2025						116,145
	Male	329	18,635	54,308	73,272	
	Female	154	10,105	36,302	46,561	
2026						129,242
	Male	314	19,585	62,782	82,681	
	Female	152	10,190	38,420	48,762	
2027						136,011
	Male	314	19,881	67,054	87,249	
	Female	154	10,447	40,803	51,404	
2028						144,467
	Male	321	20,567	72,175	93,063	
	Female	158	10,642	42,651	53,451	
2029						150,845
	Male	333	21,143	75,918	97,394	
	Female	161	10,133	37,621	47,915	
2030						135,225
	Male	350	20,282	66,678	87,310	

a: The predicted values are rounded to retain only the integer portion.

## Discussion

PD is the second most common neurodegenerative disease after Alzheimer’s disease (Aarsland et al., 2021). It is a typical chronic disease of the elderly, and the incidence of PD is increasing as life expectancy increases worldwide. In this study, we analyzed the long-term trend of PD incidence and mortality under the APC framework, and projected the PD incidence and mortality from 2020 to 2030 in China with the BAPC model, corresponding gender differences were also analyzed. To the best of our knowledge, this study is the first work to explore the long-term trends of PD incidence and mortality for the past three decades under the APC framework and project the PD incidence and mortality from 2020 to 2030 in China using the BAPC approach based on the GBD 2019 study.

We found that the PD incidence showed an upward trend from 1990 to 2019 and this trend will continue between 2020 and 2030 for both genders as projected, though males may have a higher incidence. Unlike incidence, PD mortality showed downward trends for both genders from 1990 to 2019. The projected mortality of both genders also showed downward trends across age groups from 2020 to 2030. Significant gender differences in PD incidence and mortality have been observed.

When exploring the long-term trends of disease incidence and mortality, the APC model can simultaneously control the three factors age, time period, and birth cohort. It can eliminate the interaction between the three factors, and more accurately reflect the changing trend of PD incidence and mortality in age, period, and birth cohort. Based on the results obtained from the APC analysis, we found that though the ASIRs of PD slightly increased (*P*s < 0.05) with AAPCs of 0.2% (females) and 0.8% (males), the ASMRs of PD significantly decreased from 1990 to 2019 in China (*P*s < 0.05) with AAPCs of −1.2% (females) and −0.3% (females). It is well acknowledged that age is positively associated with the initiation and progression of PD. The increase in ASIR may be largely related to the aging of Chinese society since PD is generally a disease of the elder. Furthermore, with the increasing attention paid to medication therapies, non-medication strategies (such as ablative surgeries, electric stimulations, cell therapies, and gene editing), and physiotherapies in PD, the patients might have better and longer life expectations (Domingos et al., 2018; Raza et al., 2019).

The longitudinal age curve represents the effect of social-economical and biological changing along with aging. It also suggested that the increased CIRs and CMRs were closely related to the deepening aging of China’s society. The age of 60–65 is considered the beginning of aging, which is also equivalent to the retirement age in most countries (Fereshtehnejad et al., 2014). The average age of onset of Parkinson’s disease is also around that age (van den Eeden et al., 2013). Though the etiology of PD has not been fully understood yet, however, researchers have determined that PD initiates and progresses with age (Rani et al., 2022). Published study also revealed that the dopamine (DA) neurons, which have been found to be associated with PD symptoms, decreased by 5% to 12% every 10 years as people get aged (Pan and Yue, 2014). As shown by the longitudinal age curve obtained in this study, PD incidence increased significantly after the age group of 60–64 years for both genders, which is consistent with published conclusions.

The period RRs suggested that PD incidence risk increased while PD mortality decreased for the past three decades. This may be associated with China’s socio-economic development and the continuous enrichment of social resources in recent years. The period effect is composed of the effects of various related factors and their changes which affect all age groups during the data collection period (Keyes and Li, 2012; Bell, 2020). These related factors include (but are not limited to) socioeconomic, cultural, medical resources, health awareness, lifestyle (smoking, drinking, and eating habits), and how they have changed over the data collection period (Keyes and Li, 2012). During the past several decades, the rapid development of China’s society has greatly changed people’s way of life while improved living conditions also promote health and wellness (Chiang et al., 2021). In addition, the “Notice on Strengthening Home Medical Services for the Elderly” issued by the National Health Commission of China in 2020 requires qualified medical institutions to actively provide the elderly with door-to-door medical and nursing services such as disease diagnosis and treatment, medical care, and rehabilitation (National Health Commission of P.R. China., 2020).

The cohort effect is closely related to the effect of age and time period. As shown in Figures 1D and 2, we found that younger generations (people born in the later birth cohorts) had a lower risk of PD mortality while the incidence risk increased. These results were consistent with the period effects and the results obtained in Joinpoint analysis. Our results were also consistent with studies based on other populations (Clarke, 1993; Ajdacic-Gross et al., 2012). Unhealthy lifestyles, social-economic status, education, and other factors were all found to be associated with PD (Li et al., 2019). The living conditions and health awareness of the younger generation are usually better than those of the older. These differences are partly due to better education received by the younger generation, and also to the improved living conditions (Anna et al., 2011; Xu and Shang, 2016). Besides, early-onset PD (EOPD) accounts for 3%–5% of all PD (Schrag and Schott, 2006), and the incidence of EOPD significantly increased during the past few decades (Willis et al., 2013; Ylikotila et al., 2015). However, compared to late-onset PD (LOPD), EOPD might have a more favorable prognosis (Diamond et al., 1989), which might attribute to lower PD mortality in younger generations.

In BAPC-based projection analysis, the projected age-specific PD incidence from 2020 to 2030 continues to rise for both genders. This suggested that the burden of PD for both genders of all age groups in China may continue to increase. Published review study has revealed that PD incidence is increasing with age (Tysnes and Storstein, 2017), therefore, with the deepening aging of Chinese society, the burden of PD may not decrease in the short-term. Besides, as suggested by a published study, the incidence of EOPD is increasing nowadays (Willis et al., 2013), which might also be attributed to the increased incidence of PD. However, we also found that the projected PD mortality rates for both genders continue to decrease for the next decade. In the age group 25–49, the declining trend is smooth, this may mainly be because EOPD patients had better survival prognosis (Ferguson et al., 2016). As shown in projection analysis, the age groups higher than 50, also showed a relative rapid decline in PD mortality for the next decade. In general, PD progressed with age (Rani et al., 2022), for senior aged and late-stage PD patients, death may occur due to the disease itself or comorbidities (Bloem et al., 2021; Hwang et al., 2021). However, recent development in medication and non-medication therapies in PD has greatly improved the quality of life and life expectations of PD patients (Domingos et al., 2018; Raza et al., 2019).This may be associated with the decreasing trend of mortality in late age groups.

With literature review, we found Dorsey et al. have conducted a projection study of the number of PD cases from 2005 to 2030 (Dorsey et al., 2007). Their work was based primarily on estimating and forecasting age-specific incidence and population levels in major countries (Dorsey et al., 2007). In this study, we conducted the projection analysis based on the BAPC approach and the projected number of PD patients would reach 4.787 million in 2030. Though with a different methodology, our results were consistent with Dorsey’s study (Dorsey et al., 2007). Our projection suggested that the number of individuals with PD will substantially grow over the next decade. In addition, China’s population growth rate has changed a lot in recent years. The data on which the projection analysis of this study was based would be more recent than in Dorsey’s (Dorsey et al., 2007) and closer to the current actual situation, thus our projection may be more reliable.

A significant gender difference has been observed in the incidence and mortality of PD during the past several decades, as well as in the projection analysis. A published study has suggested that PD incidence in males is higher than in females (Ascherio and Schwarzschild, 2016), in this study we also obtained the same results. Epidemiological evidence suggested that circulating estradiol may have a neuroprotective effect on the initiation of PD (Cerri et al., 2019). Females with high cumulative estrogen level during their lifetime was found to have significantly lower PD risk (Gatto et al., 2014). On the other hand, females live longer, and they have an innate tendency to be caregivers rather than care recipients, which leads to lower attention being paid to females (Cerri et al., 2019). A retrospective study also revealed that females had fewer opportunities to achieve specialist care (Willis et al., 2011). Thus, in the future prevention practice of PD and caring for PD patients, females may also need to be given more attention to maintain the downward trend of PD incidence and mortality.

In this study, we applied the BAPC analysis to project the PD incidence and mortality from 2020 to 2030. The BAPC model was a combination of the basic APC model with the Bayesian methodology. Simulation and the empirical study suggested that the combination of the two methods makes the selection of parameters and prior probability distributions more flexible, and the projection results are more robust and reliable (Møller et al., 2003; Schmid and Held, 2004; Bray and Møller, 2006). In the estimation of the BAPC model, a Markov Chain Monte Carlo (MCMC) approach was used (Riebler and Held, 2017). When using the MCMC approach to estimate the model parameters, the parameter distribution would be decided depending on the features of the data, thus the effects of age, period, and cohort can be further smoothed, avoiding large fluctuations in the estimation, and making the results more stable and reliable (Bray and Møller, 2006). Thus, the results obtained with the BAPC approach in this study were reliable.

This study also had some limitations. First, though the data obtained from GBD 2019 study is reliable, potential bias may not be avoided in the modeling process. Then, the Bayesian and classic age-period-cohort analysis both requires fixed age and time period, therefore, people aged over 95 years were removed. Besides, the analysis was conducted based on summarized data, while a cohort study based on a large population may provide more detailed results. In addition, the projection of the major clinical subtypes and the specific cause of death of PD patients in the next decade would also provide valuable information for future public health policymaking and the management of health resources. However, data about the subtypes and specific causes of death for PD patients in China was not available in the GBD 2019 database. This made us unable to project the major clinical subtypes or the cause of death of PD patients for the next decade. A population-based study focusing on these two issues may be worth to be conducted in the future.

## Conclusion

In summary, our analysis suggested that the CIRs, CMRs, and ASIRs increased in China among both males and females from 1990 to 2019, while the ASMRs decreased. Males had higher AAPCs in both PD incidence and mortality than females. Projection analysis suggested that the PD incidence will continue to increase, there may be 4.787 million PD patients in 2030 as projected. The projection analysis also indicated that mortality for both genders may be under control. The gender difference was observed in PD incidence and mortality during the past decades, as well as in projection analysis. As China’s society continues to age, enhancing the understanding of PD risk factors may help early identification of individuals at high PD risk, thereby promoting their late-life quality and effectively reducing the burden of PD.

## Data Availability Statement

Publicly available datasets were analyzed in this study. This data can be found here: The data analyzed in this study can be obtained at: http://ghdx.healthdata.org/gbd-results-tool.

## Author Contributions

FC and JM conceived and designed the study, supervised the study, and conducted critical revisions of the manuscript. FC, SC, and AS did the statistical analysis and drafted the manuscript. SC, AS, YL, WH, and YZ contributed to the acquisition, analysis, and interpretation of data. All authors contributed to the article and approved the submitted version.

## Footnotes

^1^ http://ghdx.healthdata.org/gbd-results-tool

^2^ https://analysistools.nci.nih.gov/apc/
